# Association of composite dietary antioxidant index with mortality in adults with hypertension: evidence from NHANES

**DOI:** 10.3389/fnut.2024.1371928

**Published:** 2024-05-13

**Authors:** Huali Qin, Li Shen, Danyan Xu

**Affiliations:** Department of Internal Cardiovascular Medicine, Second Xiangya Hospital, Central South University, Changsha, China

**Keywords:** CDAI, all-cause mortality, cardiovascular mortality, cancer mortality, hypertension

## Abstract

**Objective:**

The objective of this study is to assess the correlation between composite dietary antioxidant index (CDAI) with all-cause mortality and cause-specific mortality in adults with hypertension.

**Methods:**

The cohort study comprised adult participants with hypertension from the NHANES database, spanning 9 cycles from 2001 to 2018. Follow-up was conducted until December 31, 2019. Multi-variable Cox regression analysis was utilized to ascertain hazard ratios (HR) and their corresponding 95% confidence intervals, evaluating the relationship between CDAI and the risks of all-cause and cause-specific mortality. To further investigate the association between CDAI and mortality rates in adults with hypertension, Kaplan–Meier survival curves, restricted cubic splines (RCS), subgroup analyses and sensitivity analyses were employed.

**Results:**

The analysis included 16,713 adults with hypertension (mean age 56.93 ± 0.23 years, 8,327 [49.61%] male). During the mean follow-up time 102.11 ± 1.22 months, with 3,908 (18.08%) all-cause mortality occurred, 1,082 (4.84%) cardiovascular mortality and 833 (3.80%) cancer mortality. Compared to the lowest quartile of CDAI, the weighted multivariate hazard ratios of participants in the highest quartile was 0.77 (95% CI, 0.68–0.87) for all-cause mortality, 0.83 (95% CI, 0.67–1.04) for cardiovascular mortality, and 0.64 (95% CI, 0.50–0.82) for cancer mortality. RCS analysis demonstrated a nonlinear association of CDAI with all-cause and cancer mortality, and a linear association between CDAI and cardiovascular mortality. The results were robust in subgroup analyses and sensitivity analyses.

**Conclusion:**

Higher CDAI is associated with reduced all-cause mortality, cardiovascular mortality, and cancer mortality in hypertensive adults. Our findings highlight the importance of an antioxidant diet in improving outcomes in adults with hypertension.

## GRAPHICAL ABSTRACT

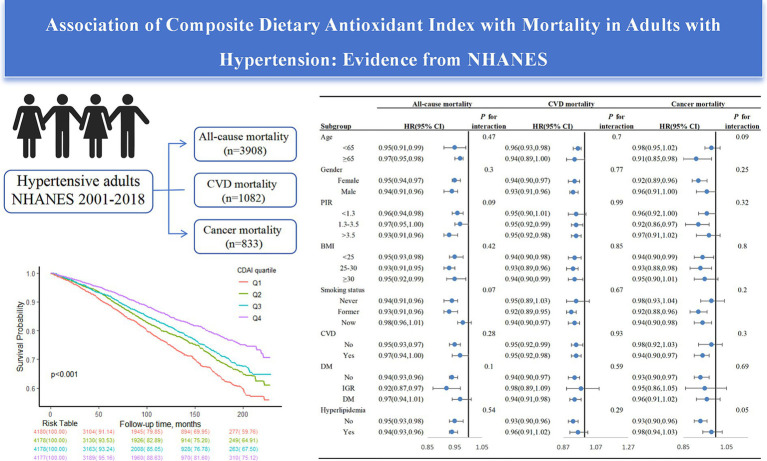

## 1 Introduction

The prevalence of hypertension is a major public health concern worldwide, affecting a significant proportion of the global population (1). The prevalence of hypertension tends to increase with aging, with approximately 1.39 billion individuals (31.1%) diagnosed with hypertension as of 2010 (2). There are several vital organs affected by this chronic condition, including the heart, cerebrovascular system, and kidneys. It is also a primary cause of cardiovascular disease-related deaths (3). Identification of reversible risk factors is therefore clinically important, as it can delay or even prevent hypertension-associated damage to target organs.

The pathogenesis of hypertension is greatly influenced by oxidative stress (4). It involves an excessive generation of reactive oxygen species (ROS) and impairment of antioxidative homeostasis, resulting in post-translational modifications of proteins and aberrant signal transduction (5). These processes contribute to endothelial dysfunction, inflammation, and vascular remodeling (4). The influence of eating patterns on cardiometabolic status has long been a significant concern in public health (6). Research findings indicate that dietary antioxidants may have a significant impact on reducing blood pressure and preventing cardiovascular disease-related mortality (7–9).

Composite Dietary Antioxidant Index (CDAI) assesses the daily intake of antioxidants, including vitamins A, C, E, zinc, selenium, and carotenoids (9). CDAI was developed with the purpose of assessing and quantifying the overall health benefits of dietary antioxidants. Study has shown that an elevated CDAI level could potentially lower the risk of death in cancer survival individuals (10). Wu et al. (8) emphasized that higher levels of CDAI reduce the risk of hypertension. However, as long-term studies are lacking, it is unclear that if CDAI are associated with a decreased hazard of overall and disease-specific mortality in those identified as having hypertension. We examined the potential correlation between CDAI and total and disease-specific mortality in adults diagnosed with hypertension using NHANES data from 2001 to 2018.

## 2 Methods

### 2.1 Study population

NHANES employs a multiple-stage, cluster sampling strategy to ensure that samples are nationally representative. Information from the NHANES study is gathered via surveys that inquire about characteristics, financial status, eating habits, and medical concerns. Each year, approximately 5,000 nationally representative participants participate in the survey. The survey protocol at the Centers for Disease Control and Prevention (CDC) has been approved by the Institutional Review Board of the National Center for Health Statistics (NCHS). Before any participant is enrolled in the study, they are required to provide written informed consent.

**Figure 1:**
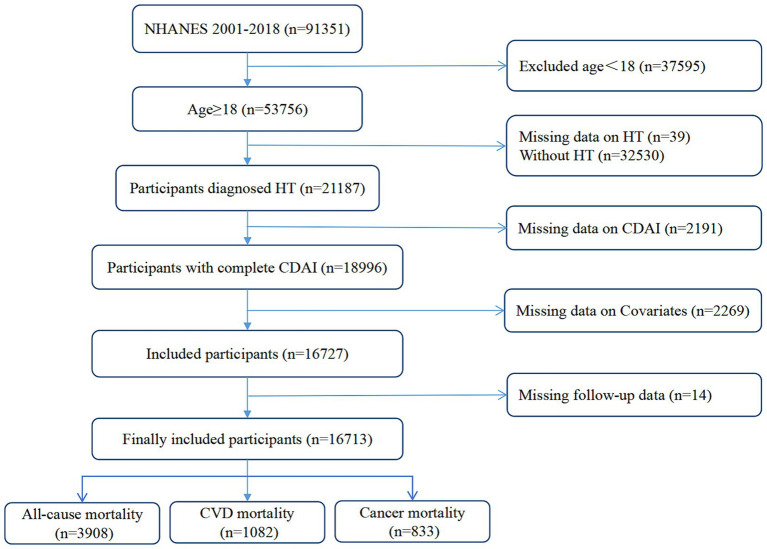
Flowchart of NHANES participants with hypertension enrolled in this study.

A nine-cycle NHANES survey spanning 2001–2018 was used in our study. We selected individuals with available data on dietary antioxidant intake, survival status, demographics and history of disease. Individuals with incomplete or unidentified information were not included in the study. In the end, 16,713 individuals aged 18 years or older with hypertension met the criteria for inclusion after applying the specified criteria for inclusion and exclusion. These participants represented 75,258,953 non-institutionalized U.S. residents. Figure 1 demonstrates the process of including and excluding participants.

### 2.2 Dietary assessment

The evaluation of CDAI utilized the index established by Wright et al. (11). A 24-h dietary recall was conducted for each participant in NHANES in order to obtain information about their dietary antioxidant intake and other food components. Dietary antioxidant intake data was obtained from NHANES participants through in-person interviews. Investigators normalized the amount six dietary antioxidants, such as vitamins A, C, E, zinc, selenium, and total carotenoids, without including supplements, drugs, or water, in order to determine the CDAI.

### 2.3 Definition of hypertension

To identify hypertension, one or more of these criteria were utilized: Patients self-report that they have hypertension or taking medications to lower blood pressure currently. The systolic and diastolic blood pressure readings are taken three times to ensure accuracy and are considered high if they exceed the standard reference range.

### 2.4 The collection of survival data

The NCHS connects data from population surveys with death certificates from the NDI to understand mortality rates and causes for specific outcomes. The term “all-cause mortality” referred to all types of deaths that occurred in this study. To identify and classify the specific subcategories of causes of death, the study relied on the NDI data and followed the coding guidelines outlined in the International Classification of Diseases. During the observation period that ended on December 31, 2019, or the date of death, a total of 3,908 deaths were recorded, out of which 1,082 were attributed to cardiovascular diseases and 833 were attributed to cancers.

### 2.5 Covariates

This study has identified various significant covariates. The data related to these covariates was collected through baseline questionnaires, which were administered by trained health technicians, interviewers, and physicians.

Covariates included: age, sex, ethnicity, marital status, level of education, poverty-to-income ratio (PIR), body mass index (BMI), energy consumption, carbohydrate consumption, dietary fiber consumption, smoking habits and tobacco exposure (serum cotinine), alcohol consumption, caffeine consumption. BMI was stratified as “<25, 25–30, or ≥30,” indicating normal weight, overweight, and obese, respectively. The classification for smoking status were based on criteria used during previous research (12). Serum cotinine was determined by liquid chromatography/mass spectrometry. CVD status was determined by self-reported diagnosis. Diabetes mellitus can be identified by meeting any of the following conditions: a prior diagnosis, HbA1C level of 6.5% or above, fasting plasma glucose ≥7.0 mmol/L, random plasma glucose level ≥11.1 mmol/L, 2 h OGTT ≥11.1 mmol/L, or taking glucose-lowering medications. Impaired Glucose Regulation is defined as exceeding the standard reference limit but not meeting the above criteria. Hyperlipidemia was defined as blood lipids exceeding the upper reference limit or using lipid-lowering drugs.

### 2.6 Statistical analysis

The data from the NHANES project were collected and processed using the nhanesR package. To ensure accurate nationwide estimates in the United States, all analyses were conducted using sample weights. Continuous variables are presented in Mean ± standard error format, and categorical variables are presented in frequency (%). One-way ANOVA was employed to analyze the continuous variables, while the χ^2^ test was used to analyze the categorical variables across the four quartiles.

To evaluate the relationship of CDAI with overall and disease-specific mortality in hypertensive adults, three Cox proportional hazards models were employed. Three models were as follows: Crude Model: This model was unadjusted. Model 1: Adjusted for age, sex, ethnicity, marital status, education level and PIR. Model 2 was modified to account for age, sex, ethnicity, marital status, level of education, PIR, caloric intake, BMI, alcohol consumption, caffeine consumption, serum cotinine, cardiovascular disease, DM, and hyperlipidemia.

We utilized restricted cubic spline models to examine the relationship between CDAI and mortality. Furthermore, we conducted subgroup analysis, interaction testing, and sensitivity analyses to evaluate the credibility of the results. Statistical analyses were conducted using R version 4.2.1 software, with significance determined by *p*-values below 0.05.

## 3 Results

### 3.1 Baseline profiles

Participants’ sociodemographic and health conditions were weighted and presented in Table 1 according to the quartiles of CDAI. The study population comprised a total of 16,713 participants with hypertension. Their average age was 56.93 ± 0.23 years, consisted of 8,327 (49.61%) male and 8,386 (50.39%) female. In comparison to people in the lowest quartile, individuals with the highest CDAI level exhibited the following characteristics: younger, more commonly male and non-Hispanic White, more educated and higher family income, and were more likely to be coupled. In terms of dietary intake, individuals in the first quartile had lower intakes of energy, dietary carbohydrates and fiber. Moreover, subjects with higher CDAI had a lower incidence of cardiovascular disease and DM. Supplementary Table S1 showed that compared with survivors, participants who died were more likely to take lower level of dietary vitamins E, zinc, selenium, and total carotenoids, while dietary vitamins A, C increased slightly but without statistically significant.

**Table 1:** Baseline characteristic of participants by CDAI levels quartiles among adults with HT in NHANES 2001–2018, weighted.

Variables	Total (*n* = 16,713)	CDAI				*p* value
		Q1 (*n* = 4,180)	Q2 (*n* = 4,178)	Q3 (*n* = 4,178)	Q4 (*n* = 4,177)	
CDAI range	(−7.06, 48.29)	(−7.06, −2.51)	(−2.51, −0.67)	(−0.67, 1.74)	(1.74, 48.29)	
Age, years	56.93 (0.23)	57.58 (0.38)	57.93 (0.38)	57.28 (0.36)	55.27 (0.36)	<0.0001
PIR	2.96 (0.03)	2.51 (0.04)	2.90 (0.04)	3.09 (0.04)	3.23 (0.04)	<0.0001
Energy, kcal/d	2071.51 (11.93)	1297.73 (12.46)	1789.88 (14.57)	2184.62 (17.38)	2788.48 (24.75)	<0.0001
Carbohydrate, g/d	244.09 (1.46)	162.87 (1.89)	214.38 (2.13)	255.36 (2.35)	320.01 (3.23)	<0.0001
Fiber, g/d	16.21 (0.14)	8.45 (0.11)	13.39 (0.14)	17.12 (0.20)	23.62 (0.25)	<0.0001
Caffeine, mg/d	180.51 (3.23)	167.76 (5.62)	179.38 (5.31)	184.88 (5.09)	187.02 (4.96)	0.02
Alcohol, g/d	11.07 (0.40)	9.66 (0.65)	9.74 (0.60)	11.69 (0.70)	12.70 (0.81)	0.01
Cotinine, ng/ml	58.84 (0.98)	72.94 (1.99)	59.31 (1.86)	56.02 (1.87)	50.46 (1.48)	<0.0001
Age, *n* (%)						<0.0001
<65	9,742 (66.30)	2,312 (64.03)	2,308 (63.21)	2,432 (65.63)	2,690 (71.26)	
≥65	6,971 (33.70)	1,868 (35.97)	1,870 (36.79)	1,746 (34.37)	1,487 (28.74)	
Gender, *n* (%)						0.14
Male	8,327 (49.61)	2,100 (47.51)	2,053 (48.73)	2,092 (50.52)	2,082 (51.10)	
Female	8,386 (50.39)	2,080 (52.49)	2,125 (51.27)	2,086 (49.48)	2,095 (48.90)	
Race, *n* (%)						<0.0001
Non-Hispanic White	7,886 (71.37)	1,752 (65.70)	1,994 (71.59)	2,076 (73.37)	2,064 (73.60)	
Non-Hispanic Black	4,304 (13.24)	1,286 (17.77)	1,063 (13.26)	955 (11.32)	1,000 (11.58)	
Mexican American	2,161 (5.55)	560 (5.72)	536 (5.51)	545 (5.48)	520 (5.52)	
Other	2,362 (9.84)	582 (10.81)	585 (9.64)	602 (9.83)	593 (9.30)	
Marital status, *n* (%)						<0.0001
Singled	6,897 (36.91)	1,904 (41.93)	1,737 (37.14)	1,657 (35.36)	1,599 (34.38)	
Coupled	9,816 (63.09)	2,276 (58.07)	2,441 (62.86)	2,521 (64.64)	2,578 (65.62)	
Education level, *n* (%)						<0.0001
<High school	4,711 (18.39)	1,599 (27.25)	1,177 (18.80)	1,053 (16.67)	882 (12.94)	
High school	4,164 (26.08)	1,062 (29.37)	1,103 (28.92)	1,006 (24.11)	993 (23.02)	
>High school	7,838 (55.53)	1,519 (43.38)	1,898 (52.28)	2,119 (59.21)	2,302 (64.04)	
PIR, *n* (%)						<0.0001
<1.3	5,189 (21.63)	1,697 (31.01)	1,284 (21.57)	1,131 (18.23)	1,077 (17.74)	
1.3–3.5	6,645 (37.53)	1,631 (39.70)	1,715 (39.61)	1,728 (38.39)	1,571 (33.35)	
>3.5	4,879 (40.84)	852 (29.30)	1,179 (38.83)	1,319 (43.38)	1,529 (48.91)	
BMI, *n* (%)						0.04
<25	3,320 (19.01)	918 (21.34)	772 (17.33)	785 (18.52)	845 (19.14)	
25–30	5,523 (32.86)	1,343 (30.71)	1,437 (34.30)	1,444 (34.34)	1,299 (31.88)	
≥30	7,870 (48.13)	1,919 (47.95)	1,969 (48.37)	1,949 (47.14)	2,033 (48.98)	
Smoking status, *n* (%)						<0.0001
Never	8,242 (49.00)	1,905 (44.82)	2,048 (48.14)	2,089 (49.28)	2,200 (52.62)	
Former	5,315 (31.83)	1,268 (29.47)	1,362 (32.52)	1,383 (32.89)	1,302 (32.05)	
Now	3,156 (19.17)	1,007 (25.70)	768 (19.35)	706 (17.83)	675 (15.33)	
CVD, *n* (%)						<0.0001
No	13,259 (82.51)	3,143 (78.01)	3,263 (81.58)	3,379 (83.39)	3,474 (85.86)	
Yes	3,454 (17.49)	1,037 (21.99)	915 (18.42)	799 (16.61)	703 (14.14)	
DM, *n* (%)						<0.0001
No	10,222 (66.26)	2,482 (63.99)	2,488 (63.98)	2,604 (66.58)	2,648 (69.62)	
IGR	1,530 (9.32)	360 (8.00)	379 (9.83)	392 (9.84)	399 (9.41)	
DM	4,961 (24.42)	1,338 (28.01)	1,311 (26.18)	1,182 (23.58)	1,130 (20.98)	
Hyperlipidemia, *n* (%)						0.14
No	3,400 (19.23)	849 (18.85)	844 (20.03)	801 (17.61)	906 (20.33)	
Yes	13,313 (80.77)	3,331 (81.15)	3,334 (79.97)	3,377 (82.39)	3,271 (79.67)	

CDAI, composite dietary antioxidant index; PIR, income-poverty-ratio; BMI, body mass index; CVD, cardiovascular disease; DM, diabetes mellitus; IGR, impaired glucose regulation.

Data are presented as the mean (SE) for continuous variables and frequencies (percentages) for categorical variables.

One-way ANOVA was used for continuous variable and χ^2^ test was used for categorical variables among the four quartiles.

### 3.2 Kaplan–Meier survival curve

**Figure 2:**
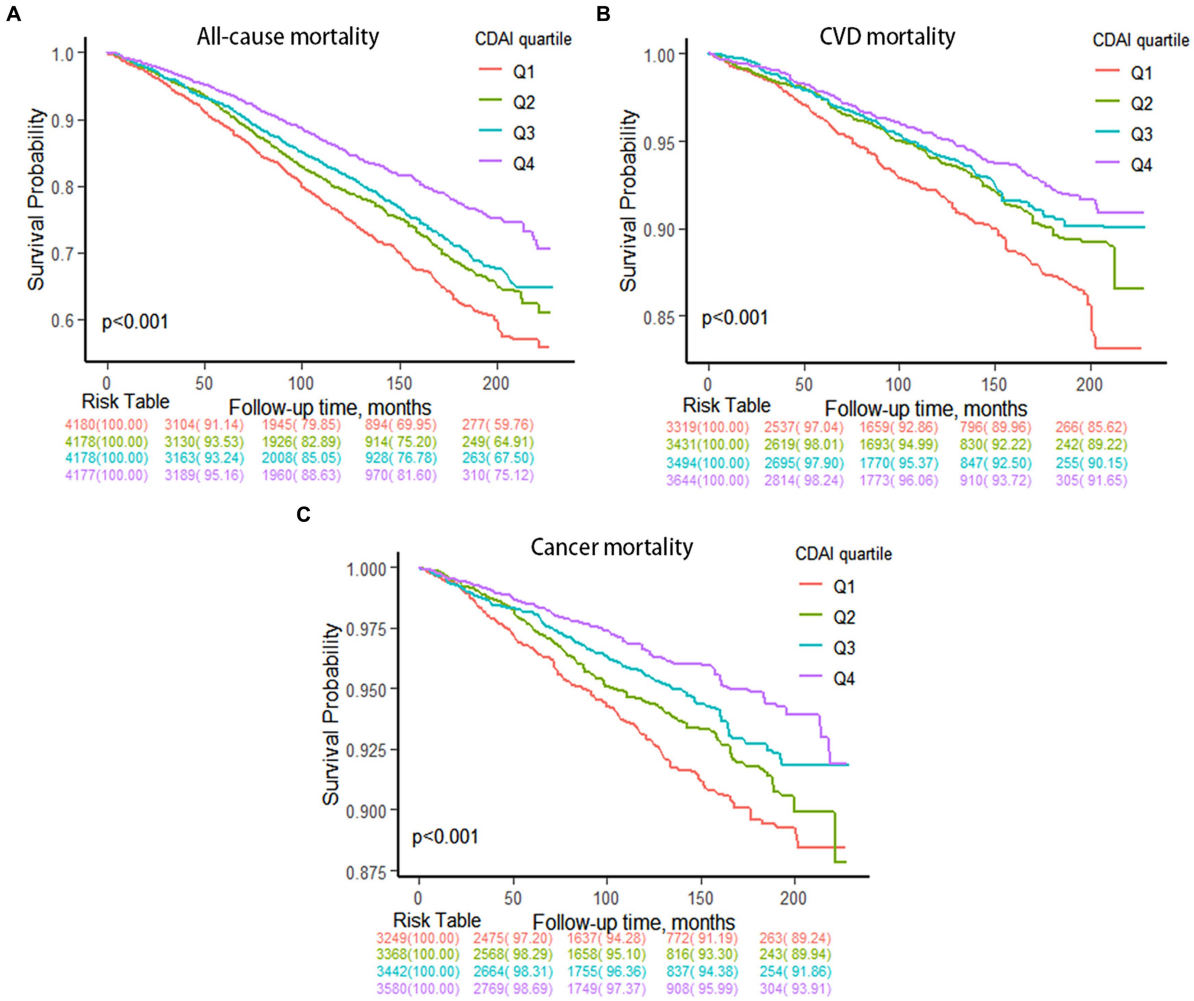
Kaplan–Meier survival curves of CDAI quartiles with mortality. **(A)** All-cause mortality. **(B)** CVD mortality. **(C)** Cancer mortality.

Among these participants, during a median follow-up period of 102.11 ± 1.22 months, there were 3,908 (18.08%) all-cause mortality occurred, 1,082 (4.84%) and 833 (3.80%) deaths specifically attributed to cardiovascular disease and cancer among adults with hypertension, respectively (Supplementary Table S2). As illustrated in Figure 2, survival was shown by weighted Kaplan–Meier survival curves in different groups according to CDAI quartiles. In comparison to Q1 (the lowest quartile), Q2, Q3, and Q4 exhibited significantly lower overall and disease-specific mortality (all *p* < 0.01). According to the Log-rank tests, subjects with lower CDAI display a tendency toward a higher risk of both all-cause and disease-specific mortality.

### 3.3 Relationship of CDAI with overall and disease-specific mortality

Table 2 and Supplementary Table S3 presented survey-weighted multivariate Cox regression results. As a continuous variable, CDAI demonstrated an inverse relationship with mortality in unadjusted models. The adjusted hazard ratios for overall mortality, cardiovascular mortality, and cancer-related mortality were 0.95, 0.94, and 0.94, respectively. In the Model 2 adjusted for all covariates, CDAI was significantly negative correlated with overall and cancer-related death, rather than CVD mortality. As a categorical variable, fully adjusted model 2 demonstrated a continued adverse correlation between CDAI and mortality in hypertensive adults. The hazards ratios for total death, CVD death, and cancer death were 0.77, 0.83, and 0.64, respectively, for participants in the highest quartile of the CDAI (Q4), as compared to those in the lowest quartile (Q1).

**Table 2:** Cox proportional hazards models for all-cause and cause-specific mortality by CDAI, weighted.

Status	HR (95% CI) *p* value				
	Q1	Q2	Q3	Q4	*P* for trend
All-cause mortality					
Crude model	Reference	0.82 (0.72,0.93) 0.002	0.74 (0.65, 0.85) <0.0001	0.55 (0.49, 0.63) <0.0001	<0.0001
Model 1	Reference	0.88 (0.77, 0.99) 0.03	0.85 (0.75, 0.98) 0.02	0.72 (0.64, 0.81) <0.0001	<0.0001
Model 2	Reference	0.88 (0.77, 1.00) 0.05	0.89 (0.78, 1.02) 0.11	0.77 (0.68, 0.87) <0.0001	<0.001
CVD mortality					
Crude model	Reference	0.74 (0.59, 0.93) 0.01	0.67 (0.54, 0.83) <0.001	0.56 (0.44, 0.72) <0.0001	<0.0001
Model 1	Reference	0.78 (0.63, 0.96) 0.02	0.74 (0.60, 0.91) 0.01	0.70 (0.56, 0.88) 0.002	0.002
Model 2	Reference	0.81 (0.64, 1.01) 0.06	0.84 (0.67, 1.04) 0.11	0.83 (0.67, 1.04) 0.10	0.13
Cancer mortality					
Crude model	Reference	0.80 (0.63, 1.02) 0.08	0.67 (0.52, 0.86) 0.001	0.50 (0.39, 0.64) <0.0001	<0.0001
Model 1	Reference	0.82 (0.64, 1.04) 0.10	0.71 (0.55, 0.92) 0.01	0.58 (0.46, 0.75) <0.0001	<0.0001
Model 2	Reference	0.84 (0.65, 1.08) 0.18	0.75 (0.58, 0.99) 0.04	0.64 (0.50, 0.82) <0.001	<0.001

CDAI, composite dietary antioxidant index; PIR, income-poverty-ratio; BMI, body mass index; CVD, cardiovascular disease; DM, diabetes mellitus; HR, hazard ratio; CI, confidence interval.

Crude model unadjusted.

Model 1 adjusted for age, gender, race, marital status, education level and PIR.

Model 2 adjusted for age, gender, race, marital status, education level, PIR, energy intake, BMI, alcohol, caffeine intake, cotinine exposure, CVD, DM, and hyperlipidemia.

**Figure 3:**
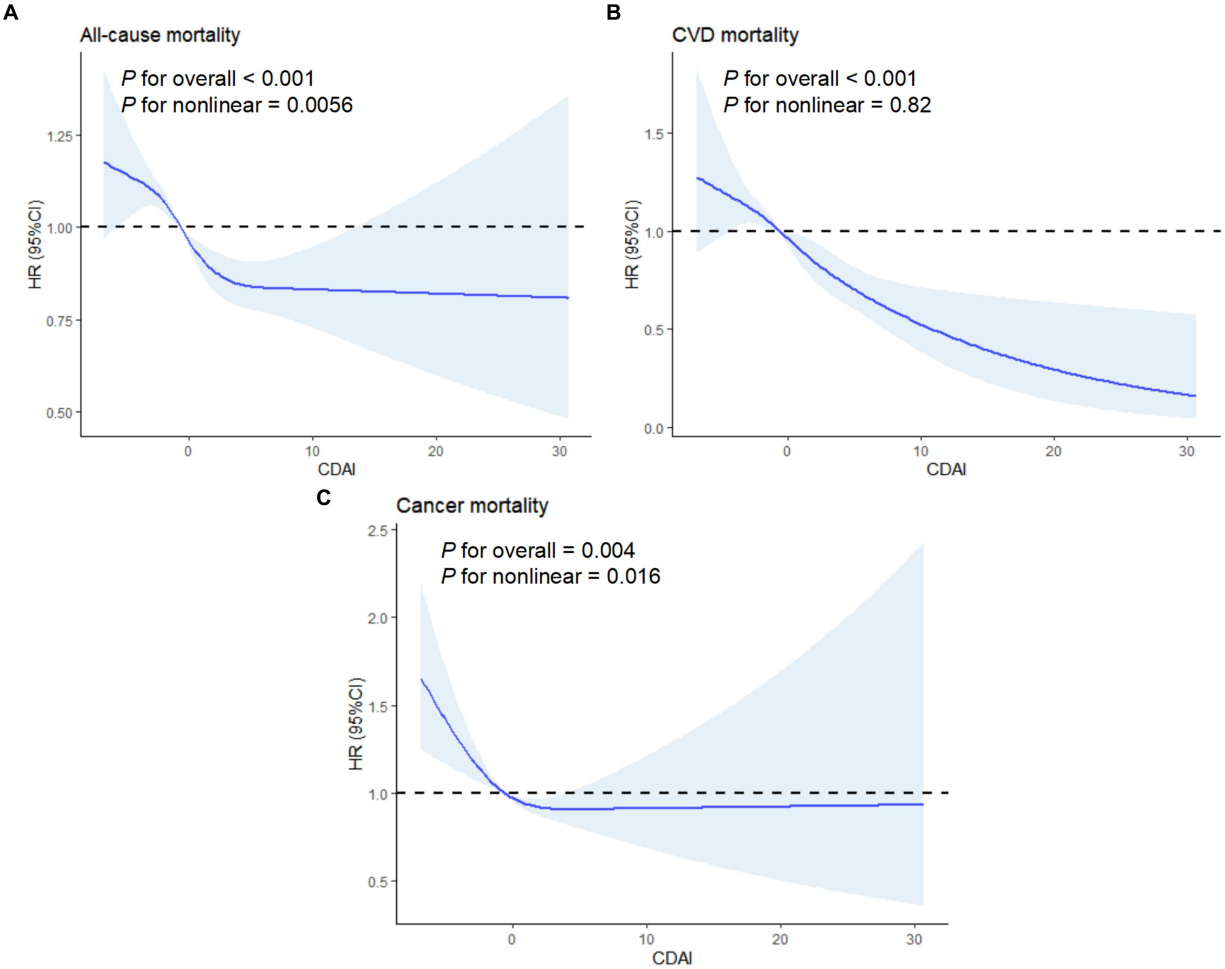
Association between CDAI and mortality using a restricted cubic spline regression model. **(A)** All-cause mortality. **(B)** CVD mortality. **(C)** Cancer mortality. Graphs show HRs for status according to CDAI adjusted for age, gender, race, marital status, education level, PIR, energy intake, BMI, alcohol, caffeine intake, cotinine exposure, CVD, DM and hyperlipidemia. Data were fitted by a restricted cubic spline Cox proportional hazards regression model. Solid lines indicate HRs, and shadow shape indicate 95% CIs. HR, hazard ratio; CI, confidence interval.

According to Figure 3, there was a clear correlation between CDAI and mortality that follows a dose-dependent pattern. It’s worth noting that a nonlinear connection between CDAI and both all-cause (*p* < 0.001, P for nonlinear = 0.0056) and cancer mortality (*p* = 0.004, P for nonlinear = 0.016). Furthermore, the correlation between CDAI and CVD mortality was more closely aligned with a linear pattern.

### 3.4 Relationship of CDAI components with overall and disease-specific mortality

Multivariate Cox regression analysis revealed the effects of CDAI components on overall and disease-specific mortality in hypertensive adults (Table 3). The weighted hazard ratios of vitamin E was found to be 0.98 for overall mortality, 0.97 for cardiovascular mortality, and 0.96 for cancer-related mortality.

**Table 3:** Effects of CDAI components on all-cause and cause-specific mortality by multivariate cox proportional hazards regression analysis, weighted.

Components		HR (95% CI) *p* value	
	All-cause mortality	Cardiovascular mortality	Cancer mortality
Vitamin A	1.00 (1.00, 1.00) 0.43	1.00 (1.00, 1.00) 0.30	1.00 (1.00, 1.00) 0.10
Vitamin C	1.00 (1.00, 1.00) 0.004	1.00 (1.00, 1.00) 0.03	1.00 (1.00, 1.00) 0.31
Vitamin E	0.98 (0.97, 0.99) <0.0001	0.97 (0.95, 0.98) <0.001	0.96 (0.94, 0.99) <0.001
Zinc	0.99 (0.98, 1.00) 0.03	0.99 (0.98, 1.00) 0.12	0.99 (0.97, 1.00) 0.28
Selenium	1.00 (0.99, 1.00) 0.02	1.00 (0.99, 1.00) 0.39	1.00 (0.99, 1.00) 0.01
Carotenoids	1.00 (1.00, 1.00) 0.02	1.00 (1.00, 1.00) 0.85	1.00 (1.00, 1.00) 0.71

HR, hazard ratio; CI, confidence interval.

Fully adjusted for age, gender, race, marital status, education level, PIR, energy intake, BMI, alcohol, caffeine intake, cotinine exposure, CVD, DM, and hyperlipidemia.

**Figure 4:**
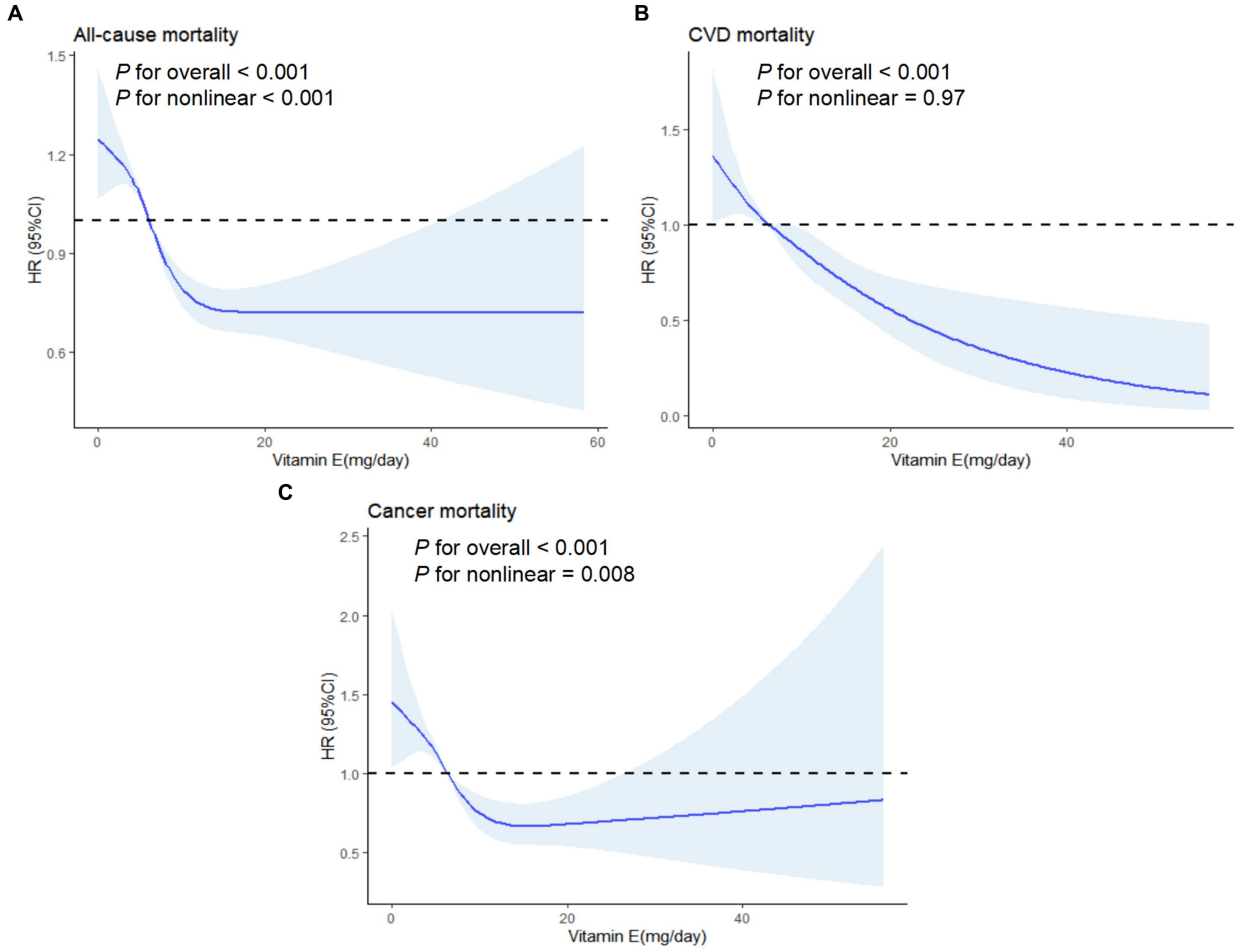
Association between vitamin E and mortality using a restricted cubic spline regression model. **(A)** All-cause mortality. **(B)** CVD mortality. **(C)** Cancer mortality. Graphs show HRs for status according to CDAI adjusted for age, gender, race, marital status, education level, PIR, energy intake, BMI, alcohol, caffeine intake, cotinine exposure, CVD, DM and hyperlipidemia. Data were fitted by a restricted cubic spline Cox proportional hazards regression model. Solid lines indicate HRs, and shadow shape indicate 95% CIs. HR, hazard ratio; CI, confidence interval.

To further explore the relationship of vitamin E with overall and disease-specific mortality, RCS analysis was conducted. Figure 4 illustrated that an inverse linear correlation between vitamin E and CVD mortality (*p* < 0.001, *P* for nonlinear = 0.9711). Additionally, the association of vitamin E between overall and cancer-related mortality displayed a nonlinear pattern (*p* < 0.001, *P* for nonlinear < 0.05). These findings suggest that certain dietary antioxidants, particularly Vitamin E may have an impact on reducing the likelihood of all-cause and cause-specific mortality in hypertensive adults.

### 3.5 Subgroups analyses and sensitivity analyses

**Figure 5:**
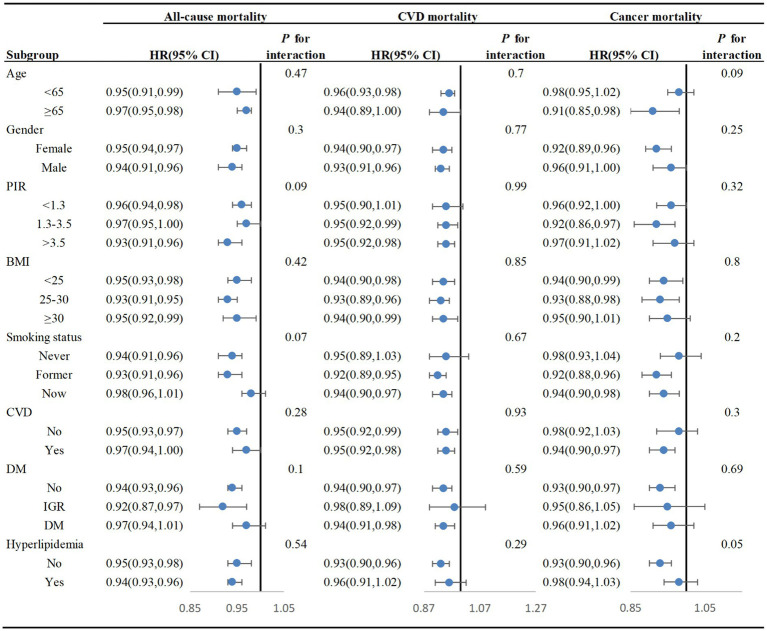
Subgroup analyses of the relationship between CDAI and mortality among adults with HT in NHANES 2001–2018, weighted. Cox proportional hazards models adjusted for age, gender, race, marital status, education level, PIR, energy intake, BMI, alcohol, caffeine intake, cotinine exposure, CVD, DM, and hyperlipidemia. In the subgroup analysis, the model is not adjusted for age, gender, poverty-to-income ratio (PIR), body mass index (BMI), cotinine exposure, cardiovascular disease (CVD), diabetes mellitus (DM), and hyperlipidemia, respectively.

In subgroup analyses, we noticed that CDAI had a stronger negative correlation with all-cause mortality in most subgroups except current smokers, CVD and DM participants. For cause-specific mortality, CDAI was negatively linked with cardiovascular and cancer-related mortality in female, non-obese, smokers and CVD participants (Figure 5). It is worth mentioning that there were no notable interactions detected among the subgroup variables (all *P* for interaction >0.05). These findings suggest that the effect of CDAI on mortality in adults with hypertension is consistent across various demographic and health-related subgroups, with no significant influence from these variables.

Two sensitivity analyses were performed to assess robustness, as detailed in Supplementary Table 4. After excluding participants who deceased within the first 2 years of follow-up, the relationship between CDAI and mortality from all causes, CVD, and cancer was found to be consistent with the findings in Table 2.

## 4 Discussion

This observational cohort study examined the relationship between dietary antioxidants and mortality rates in hypertensive adults in the United States. The results showed a significant association between the consumption of antioxidants, specifically CDAI and dietary Vitamin E, and both all-cause and cause-specific mortality. Even in the fully adjusted Cox model, higher CDAI levels were found to be linked to lower risks of mortality. The findings suggest that increasing intake of dietary antioxidants, especially Vitamin E, could potentially reduce mortality rates among individuals with hypertension. These results underscore the importance of including antioxidant-rich foods in the diets of hypertensive individuals to enhance health outcomes and mitigate mortality risks.

The CDAI functions as a thorough instrument to evaluate the diet’s overall antioxidant level, enabling the identification and categorization of possible sources of antioxidants derived from intricate dietary elements (11). This allows for classifying individual dietary patterns based on antioxidant consumption, promoting the development of healthier dietary habits and lifestyles. Previous research has documented connections between antioxidant intake and the occurrence of hypertension (8, 13, 14). Some studies have utilized NHANES data to investigate the correlation between CDAI and mortality across various populations. Wang et al.’s research indicates that elevated levels of CDAI in the general population are linked to a substantial reduction in the likelihood of both overall and CVD-related death (15). Specifically, in contrast to the Q1 group, the Q4 group showed a 10% decrease in the risk of overall mortality and a 19% reduction in CVD death. Among individuals with diabetes, those in the highest CDAI quartile experienced a 53% reduction in both overall and CVD mortality (12, 15). Furthermore, higher CDAI levels in patients with early-stage CKD were linked to a notable decrease in the risk of all-cause death (16). Xu highlighted the protective impact of CDAI on individuals suffering from post-stroke depression, as well as the favorable influence of CDAI on the recovery outlook of stroke survivors (17). A recent survey has also highlighted the inverse correlation between CDAI levels and poor prognosis in patients with osteoarthritis (18). Studies observed significant inverse correlation between consuming antioxidants like carotenoids and vitamin E and decreased risk of death from heart disease and overall mortality (19, 20). In line with prior research, our study also revealed that higher level of CDAI was linked to reduced hazards of mortality from all causes and specific causes in adults with hypertension. Maintaining a balanced and adequate dietary intake of antioxidants, including Vitamin E, is crucial for optimizing health outcomes in this population.

There are many factors involved in the etiology of hypertension (21). Oxidative stress notably in a similar way plays a vital part in developing hypertension (4, 22). ROS produced in vascular smooth muscle cells (VSMCs) contribute to oxidative stress, resulting in peroxidation of lipids and proteins, DNA damage, and the initiation of a transition in the VSMC phenotype from a contractile to a synthetic state (23, 24). These mechanisms contribute to the pathological remodeling of arteries, potentially worsening hypertension. In summary, the interaction of these elements forms a intricate network of mechanisms that contribute to the onset and development of hypertension. Grasping these processes is vital in devising effective strategies for preventing and treating this prevalent public health issue.

Previous studies has revealed a positive correlation between biomarkers of oxidative stress, including methylmalonic acid (MMA) and malondialdehyde (MDA), and the likelihood of cardiovascular disease (CVD) and hypertension (25, 26). On the contrary, dietary antioxidants, widely present in various diets, possess the capability to strengthen the body’s defense mechanisms against oxidative stress. They achieve this by scavenging surplus free radicals, thereby diminishing oxidative stress. Additionally, the close association between chronic low-grade inflammation and hypertension development is evident, given its contribution to arterial stiffness and pathological remodeling (22). Elevated levels of CDAI have been shown to significantly reduce levels of pro-inflammatory cytokines (27). Hence, dietary antioxidants can alleviate the disturbance of tissue homeostasis induced by oxidative stress and decrease the infiltration of inflammatory factors. This presents potential advantages for preventing and treating hypertension. This partly explained the negative relationship between CDAI and total and disease-specific mortality in adults with hypertension. Through the reduction of oxidative stress and inflammation, dietary antioxidants may significantly contribute to promoting cardiovascular health and enhancing outcomes for individuals managing hypertension. Numerous studies underscore a noteworthy correlation between the consumption of essential trace elements, including zinc, iron, copper, and selenium, and the likelihood of developing hypertension (28–30). Additionally, a recent cross-sectional study focusing on 5,067 women revealed a significant negative correlation between hypertension and dietary antioxidant intake (31).

Based on the rigorous standardized procedures and quality control measures of NHANES, our study’s findings can be applied to 75,258,953 non-institutionalized US residents. By incorporating nutritional factors, specifically CDAI into the realm of chronic disease management, we examined the association between CDAI and overall mortality, as well as CVD and cancer related death among individuals with hypertension. While conventional medical research has primarily focused on pharmaceutical treatments and clinical interventions, our study underscores the significance of dietary antioxidants in mitigating mortality risk within this specific demographic. Moreover, our analysis uncovers intricate relationships between specific dietary antioxidants and mortality from different causes, resulting in a more comprehensive understanding and a foundation for creating customized preventive measures. By delving into the distinct roles of various antioxidants in lowering mortality risk, our study provides practical guidance for future nutritional interventions, enabling healthcare professionals and patients to make more informed dietary choices. Through subgroup analysis and interaction detection, we further explored the impact of CDAI on mortality risk across different subgroups, confirming the robustness and applicability of our findings. In essence, our research underscores the crucial role of antioxidant-rich diets in reducing overall mortality and cause-specific deaths among hypertensive individuals, contributing valuable insights to public health initiatives and clinical practices in hypertension management.

Our study has some limitations. Firstly, in this observational cohort study, we can only analyze the correlation between dietary antioxidant intake and the risk of all-cause and cause-specific mortality in individuals with hypertension, and causality cannot be determined. Secondly, the reliance on self-reported data for both dietary questionnaires and disease status, as obtained from the NHANES database, may introduce recall bias and discrepancies between reported and actual conditions. Although NHANES employs rigorous data collection protocols, there remains a possibility of measurement error in the self-reported information. Thirdly, as our study comprises only Americans and excludes special groups such as minors, we are unable to conduct specific subgroup analyses for other ethnicities or diverse populations due to the constraints of sample size and representativeness. In order to thoroughly comprehend the connection between CDAI and the hazards of overall mortality as well as mortality from specific causes among people with hypertension, it is crucial to confirm our findings through larger prospective studies.

## 5 Conclusion

In summary, this research discovered that hypertensive adults with high CDAI levels had a lower risk of overall mortality as well as mortality from specific causes. These results established a crucial groundwork for comprehending the possible role of dietary antioxidants in managing hypertension and reducing mortality risk. Additional research will be essential to validate the observed associations and ascertain potential therapeutic implications for individuals grappling with hypertension.

## Data availability statement

The National Health and Nutrition Examination Survey dataset is publicly available at the National Center for Health Statistics of the Center for Disease Control and Prevention (https://www.cdc.gov/nchs/nhanes/index.htm). The mortality data were sourced from the National Death Index (NDI) database (https://www.cdc.gov/nchs/data-linkage/mortality-public.htm).

## Ethics statement

The studies involving humans were approved by The National Center for Health Statistics Research Ethics Review Board. The studies were conducted in accordance with the local legislation and institutional requirements. Written informed consent for participation in this study was provided by the participants' legal guardians/next of kin.

## Author contributions

HQ: Writing – original draft, Visualization, Methodology, Investigation, Formal analysis, Data curation, Conceptualization. LS: Writing – review & editing, Supervision, Funding acquisition. DX: Writing – review & editing, Supervision, Funding acquisition.
