# Supplementary material for: Association of composite dietary antioxidant index with mortality in adults with hypertension: evidence from NHANES
Source: Front Nutr. 2024 May 13;11:1371928. doi: 10.3389/fnut.2024.1371928 (PMC11132182; doi:10.3389/fnut.2024.1371928)
Supplement: Supplementary file 1 [file Table_1.docx]

# Association of Composite Dietary Antioxidant Index with Mortality in Adults with Hypertension: Evidence from NHANES

Running title: CDAI and mortality in hypertension

Huali Qin^1^, Li Shen ^1*^, Danyan Xu ^1*^

^1^ Department of Internal Cardiovascular Medicine, Second Xiangya Hospital, Central South University, Changsha, Hunan, China. 410011

^*^Corresponding author:

Li Shen (E-mail address: shenli19861222@csu.edu.cn)

Danyan Xu (E-mail address: xudanyan02@csu.edu.cn)

139 Middle Renmin Road

Department of Internal Cardiovascular Medicine

Second Xiangya Hospital

Central South University, Changsha, Hunan, China. 410011

Tel: +86 731 85292100

Fax: +86 731 85295407

This work was supported by the National Nature Scientific Funding of China (No.82072555) ，Chinese Cardiovascular Association-Access fund（2019-CCA-ACCESS-023）, and the National Nature Scientific Funding of Hunan Province and Changsha（2021JJ30948, 2021JJ3075, kq2004162, 202203013201）

**Supplementary table 1.** Level of CDAI and its components by mortality status among adults with HT in NHANES 2001–2018, weighted.

| **Variables** | **Total**  **(n=16713)** | **Status** | | ***P* value** |
| --- | --- | --- | --- | --- |
|  |  | **Survivor**  **(n=12805)** | **Death**  **(n=3908)** |  |
| CDAI | 0.36(0.05) | 0.51(0.05) | -0.32(0.09) | < 0.0001 |
| Vitamin A (μg/day) | 619.35(6.85) | 619.67( 7.42) | 617.90(14.79) | 0.91 |
| Vitamin C (mg/day) | 82.15(1.24) | 82.78(1.40) | 79.27(1.72) | 0.08 |
| Vitamin E (mg/day) | 8.07(0.09) | 8.37(0.10) | 6.70(0.12) | < 0.0001 |
| Zinc (mg/day) | 11.41(0.12) | 11.59(0.12) | 10.61(0.22) | < 0.0001 |
| Selenium (μg/day) | 109.62(0.72) | 112.52(0.77) | 96.51(1.53) | < 0.0001 |
| Carotenoids (μg/day) | 9643.45(161.04) | 9877.09(176.69) | 8584.80(288.70) | < 0.0001 |

HT hypertension, CDAI composite dietary antioxidant index;

Data are presented as the mean (SE) for continuous variables.

T-test was used for continuous variable.

**Supplementary table 2.** Mortality status of participants by CDAI levels quartiles among adults with HT in NHANES 2001–2018, weighted.

| **Variables** | **Total**  **(n=16713)** | **CDAI** | | | | ***P***  **value** |
| --- | --- | --- | --- | --- | --- | --- |
|  |  | **Q1**  **(n=4180)** | **Q2**  **(n=4178)** | **Q3**  **(n=4178)** | **Q4**  **(n=4177)** |  |
| **Follow-up time, months** | 102.11(1.22) | 102.54(2.20) | 100.69(1.64) | 101.70(1.64) | 103.37(1.71) | 0.54 |
| **Status, n(%)** |  |  |  |  |  | < 0.0001 |
| Alive | 12805(81.92) | 2988(76.07) | 3161(80.97) | 3236(82.47) | 3420(86.62) |  |
| Deceased | 3908(18.08) | 1192(23.93) | 1017(19.03) | 942(17.53) | 757(13.38) |  |
| **Leading cause, n(%)** |  |  |  |  |  | < 0.0001 |
| CVD disease | 1082( 4.84) | 330(6.50) | 270(4.83) | 258(4.46) | 224(3.96) |  |
| All other causes | 1020( 4.74) | 313(6.23) | 291(5.52) | 226(4.26) | 190(3.40) |  |
| Cancer | 833( 3.80) | 260(5.10) | 207(4.17) | 206(3.51) | 160(2.77) |  |
| Cerebrovascular diseases | 218( 0.92) | 59(1.03) | 63(0.90) | 58(1.21) | 38(0.58) |  |
| Chronic lower respiratory diseases | 203( 1.09) | 78(1.85) | 54(1.22) | 46(1.04) | 25(0.47) |  |
| DM | 148( 0.79) | 36(0.81) | 37(0.73) | 41(1.09) | 34(0.57) |  |
| Alzheimer's disease | 144( 0.71) | 40(0.85) | 28(0.57) | 43(0.79) | 33(0.67) |  |
| Kidney disease | 101( 0.39) | 33(0.60) | 20(0.29) | 23(0.36) | 25(0.33) |  |
| Accidents | 85( 0.50) | 28(0.76) | 24(0.48) | 16(0.34) | 17(0.47) |  |
| Influenza and pneumonia | 73( 0.29) | 14(0.19) | 23(0.33) | 25(0.47) | 11(0.17) |  |

HT hypertension, CDAI composite dietary antioxidant index, CVD cardiovascular disease, DM diabetes mellitus;

Data are presented as the mean (SE) for continuous variables and frequencies(percentages) for categorical variables.

One-way ANOVA was used for continuous variable and χ2 test was used for categorical variables among the four quartiles.

**Supplementary table 3.** Cox proportional hazards models for all-cause and cause-specific mortality of CDAI among adults with HT in NHANES 2001–2018, weighted.

| **Status** | **Crude model** | | **Model 1** | | **Model 2** | |
| --- | --- | --- | --- | --- | --- | --- |
|  | **HR(95%CI)** | ***P* value** | **HR(95%CI)** | ***P* value** | **HR(95%CI)** | ***P* value** |
| **All-cause mortality** | 0.95(0.93,0.96) | <0.0001 | 0.97(0.96,0.99) | <0.001 | 0.98(0.97,1.00) | 0.02 |
| **CVD mortality** | 0.94(0.91,0.96) | <0.0001 | 0.96(0.94, 0.99) | 0.002 | 0.98(0.96,1.00) | 0.07 |
| **Cancer mortality** | 0.94(0.91,0.97) | <0.0001 | 0.96(0.93,0.99) | 0.01 | 0.97(0.94,1.00) | 0.04 |

CDAI composite dietary antioxidant index, PIR income-poverty-ratio, BMI body mass index, CVD cardiovascular disease, DM diabetes mellitus, HR hazard ratio, CI confidence interval;

Crude model unadjusted.

Model 1 adjusted for age, gender, race, marital status, education level and PIR.

Model 2 adjusted for age, gender, race, marital status, education level, PIR, energy intake, BMI, alcohol, caffeine intake, cotinine exposure, CVD, DM and hyperlipidemia.

**Supplementary table 4.** Sensitivity analyses for the association of CDAI with mortality among adults in NHANES 2001–2018, weighted.

|  | **OR(95%CI)** | | | | |
| --- | --- | --- | --- | --- | --- |
|  | **Q1** | **Q2** | **Q3** | **Q4** | **P for trned** |
| **All-cause mortality** | | | | | |
| Excluded individuals who died within 1 years of follow-up | Reference | 0.89(0.78,1.01) | 0.90(0.78,1.04) | 0.76(0.67,0.86) | <0.001 |
| Excluded individuals who died within 2 years of follow-up | Reference | 0.88(0.77,1.01) | 0.89(0.77,1.03) | 0.76(0.67,0.86) | <0.001 |
| **CVD mortality** | | | | | |
| Excluded individuals who died within 1 years of follow-up | Reference | 0.78(0.63, 0.96) | 0.74(0.60,0.91) | 0.70(0.56,0.88) | 0.04 |
| Excluded individuals who died within 2 years of follow-up | Reference | 0.76(0.60,0.97) | 0.88(0.69,1.12) | 0.83(0.67,1.02) | 0.04 |
| **Cancer mortality** | | | | | |
| Excluded individuals who died within 1 years of follow-up | Reference | 0.84(0.65,1.08) | 0.75(0.58,0.99) | 0.64(0.50,0.82) | 0.19 |
| Excluded individuals who died within 2 years of follow-up | Reference | 0.84(0.64,1.10) | 0.71(0.52,0.96) | 0.62(0.47,0.81) | 0.14 |

CDAI composite dietary antioxidant index, PIR income-poverty-ratio, BMI body mass index, CVD cardiovascular disease, DM diabetes mellitus.

Cox proportional hazards model adjusted for age, gender, race, marital status, education level, PIR, energy intake, BMI, alcohol, caffeine intake, cotinine exposure, CVD, DM and hyperlipidemia.
